# Draft Genome Sequence of *Mycobacterium brumae* ATCC 51384

**DOI:** 10.1128/genomeA.00237-16

**Published:** 2016-04-28

**Authors:** Giuseppe D'Auria, Eduard Torrents, Marina Luquin, Iñaki Comas, Esther Julián

**Affiliations:** a Área de Genómica y Salud, Fundación para el Fomento de la Investigación Sanitaria y Biomédica de la Comunidad Valenciana (FISABIO-Salud Pública), Valencia, Spain; b CIBER en Epidemiología y Salud Pública (CIBEResp), Madrid, Spain; c Bacterial Infections and Antimicrobial Therapy Group, Institute for Bioengineering of Catalonia (IBEC), Barcelona, Spain; d Departament de Genètica i de Microbiologia, Facultat de Biociències, Universitat Autònoma de Barcelona, Bellaterra, Barcelona, Spain; e Instituto Biomedicina, CSIC, Valencia, Spain

## Abstract

Here, we report the draft genome sequence of *Mycobacterium brumae* type strain ATCC 51384. This is the first draft genome sequence of *M. brumae*, a nonpathogenic, rapidly growing, nonchromogenic mycobacterium, with immunotherapeutic capacities.

## GENOME ANNOUNCEMENT

*Mycobacterium brumae* is an environmental mycobacterium that was described for the first time in 1993 when it was isolated from water and soil samples and from a human sputum of an asymptomatic individual (1). Colonies on agar media are eugonic, rough, and nonpigmented. Growth occurs within 5 days at 30°C and 37°C. In liquid medium, *M. brumae* forms clumps with cording morphology (2). The cell wall of *M. brumae* contains only α-mycolic acids, which only release docosanoate after pyrolysis (1). Until now, no attention has been paid to this mycobacterium species, because no cases of infection due to *M. brumae* have been described in animals or humans. In 2004, Han et al. reported a catheter-infection case related to this species (2), but it was later confirmed that the isolate strain was not *M. brumae* (3).

We recently demonstrated the antitumor and immunomodulatory activity of this mycobacterium (4, 5). *In vitro* studies have shown the capacity of *M. brumae* to activate macrophages. *M. brumae* is able to inhibit the proliferation of bladder cancer cells, with a higher growth inhibition capacity than *M. bovis* BCG in low-grade bladder tumor cells (4). *M. brumae* triggers an antitumor profile in *ex vivo* infected peripheral blood cells, and *in vivo* studies have demonstrated that both live and γ-irradiated *M. brumae* are able to prolong survival of tumor-bearing mice (4, 5). Due to the interest in finding the antigen(s) responsible for these activities, we aimed to sequence the *M. brumae* genome.

*M. brumae* ATCC 51384 was grown on Middlebrook 7H10 medium for 1 week. Cells were scraped, and genomic DNA was isolated using the Ultra-Clean microbial DNA isolation kit (MO BIO Laboratories, Inc.) according to the manufacturer’s specifications. We sequenced the genomic DNA on an Illumina MiSeq system and assembled the reads using MIRA (6), with manual correction assisted by the STADEN package (7). A total of 3,784,633 reads were used after quality assessment and trimming, with an average read length of 361.98 bp (SD = 98.58). The assembled sequences of *M. brumae* comprised 75 contigs larger than 1.5 kb, with a combined length of 4,026,006 bp and a mean G-C ratio of 69.1%. The average and median contig sizes were 53,680 and 27,753 bp, respectively, and the longest contig was 572,857 bp. The number of predicted protein coding genes is 3,823 after RAST prediction of open reading frames (8). Average nucleotide comparison with all draft, complete, and some selected Sequence Read Archive mycobacterial genomes (April 2015, *n* = 110) corroborated that *M. brumae* forms a distinct and separate species with a minimum of 70.31% (*Mycobacterium abscessus* M94) and a maximum of 76.53% nucleotide identity (*Mycobacterium avium* subsp. *paratuberculosis* S5) with the rest of the mycobacteria species analyzed.

In conclusion, we report the genome sequence of *M. brumae* ATCC 51384 that to the best of our knowledge is the first genome sequence of this species*.*

### Nucleotide sequence accession numbers.

This whole-genome shotgun project has been deposited in European Nucleotide Archive (ENA) (http://www.ebi.ac.uk/ena) under the accession numbers FJNX00000001 to FJNX00000075. The versions described in this paper are the first versions, FJNX01000001 to FJNX01000075.
